# Hospital Finance at Its Best

**Published:** 1907-11-16

**Authors:** 


### HOSPITAL ADMINISTRATION.

### CONSTRUCTION AND ECONOMICS.

### HOSPITAL FINANCE AT ITS BEST.

GUY'S HOSPITAL AND ITS TREASURER.

In The Hospital of August 17, 1907, page 537, we gave a full account of the spending of half a million of money in the rebuilding of the London Hospital. To-day we propose to endeavour to bring out clearly the extraordinary services rendered to Guy's Hospital by Mr. H. Cosmo Bonsor, the treasurer, during the twelve years he has held office. A great hospital, such as Guy's is to-day, is, in reality, an enormous business proposition. Much as foreigners may admire the results obtained under the voluntary system of our hospitals, their appreciation would be immeasurably increased were it possible to bring home to their minds the full extent of the services which a few individual men have rendered to the business of charity, by devoting much of their energy and time, without fee or reward, to the administration of our great general hospitals. Mr. H. Cosmo Bonsor is primarily a man of business, and the importance of having a man of business as treasurer of a great hospital like Guy's is provable by the history of that institution during the last twelve years. In this period the whole of the older portions of the buildings have been reconstructed and modernised; every department has been thoroughly overhauled; new features have been introduced, and blocks of new buildings have been erected with so much judgment and foresight that, when the whole work is completed, Guy's Hospital may be regarded as one of the most efficient and up to date of modern hospitals.

Strictly Business Methods.

As a man of business Mr. Bonsor appears to have made it his first consideration so to organise the work as to secure three great results: (1) that guarantees should be forthcoming that every pound expended should produce a pound's result in value; (2) that the cost of the whole of the reconstruction and new buildings should be defrayed out of moneys actually raised for this purpose at a minimum cost, and (3) that not one penny of the money should be taken from capital, that is, from the realisation of investments or reserve funds. With the object of securing the strictest economy and the fullest possible return on the capital outlay, Mr. Cosmo Bonsor associated with himself Sir Cooper Perry, M.D., as the superintendent of the hospital. Sir Cooper Perry's university career stamps him as one of the intellectual forces of his day; he has made hospital administration and construction matters of earnest study, so that he is one of the most knowledgeable hospital superintendents in the world. Mr. Cosmo Bonsor determined too that the whole of the building and reconstruction work should be carried out under their direction by the hospital's own surveyor and staff. Mr. J. H. T. Woodd was appointed to the latter office, and the whole of the planning and building arrangements have been executed and carried out by Sir Cooper Perry and the surveyor, with results which make them memorable in the history of hospital reconstruction.

A Great Saving in Cost.

It is difficult to say exactly the amount of actual cash which has been saved by the adoption of this system, but on a low computation it cannot be less, we imagine, than £60,000. In other words, the new buildings and reconstruction of the hospital have been completed for a sum of about £350,000, instead of entailing an expenditure of upwards of £400,000, as they otherwise certainly would have done. The system pursued has the further advantage that every part of the reconstruction works and every new building has been perfected as to its details, before any portion of it was commenced. By this means a further considerable sum has been saved, for nothing is more costly than to modify or change the plans of a building, after it has been commenced. We take entire responsibility for the estimate of the savings in expenditure thus effected at Guy's Hospital, knowing it to be based upon an exceptional experience of building operations, extending over a period of many years.

£350,000 Raised and Spent.

The next advantage which has accrued to Guy's Hospital from the possession of a treasurer who is a good man of business has been the defraying of the whole of the actual expenditure, without the outlay of a single shilling of the hospital's capital funds. The scheme now completed and paid for presents another remarkable feature, for so quietly has the appeal work been carried on, that the public have heard very little about it. We have no doubt that the mention of the vast figures involved will create general surprise. Further, and more important still, the quiet, steady, persistent appeal work, successfully accomplished under Mr. Cosmo Bonsor's direction by Mr. C. H. Wells, the secretary to the treasurer, has entailed an expenditure so small as to amount to less than five per cent. upon the total sum represented by the donations, subscriptions and other voluntary contributions. Thus the total outlay during the twelve years for salaries, permanent and temporary clerks, and commissions of all kinds amounts to but 3.1 per cent., whilst the outlay on printing, stationery, advertising, postage, festivals and every other item of the kind represents only 1.8 per cent. of the total receipts. Hence nearly £350,000 has been raised at a cost of only 4.9 per cent. We believe this result to be unique in the history of the voluntary hospitals of this country.

The Extent of the Building Operations.

The vast extent of the building operations will be readily seen when we state that, in addition to extensive and necessary repairs to bring the old buildings to a state of thorough efficiency, all the wards in the old buildings have been renovated and modernised, and new sanitary blocks have been provided throughout the whole hospital. These renovations have entailed the re-flooring of the wards, the addition of modern and perfected operation theatres, the provision of new window frames specially designed to afford the maximum of ventilation without draught, the replastering of most of the walls, and the addition of a multitude of devices in wood for the reception of stores, instruments, the various scientific and other apparatus required for the work in each ward, the sterilisation of dressings and their preservation in a condition to keep them efficient for immediate use, and very many other kinds of equipment. Nearly all the wards have been provided with open-air balconies, and most of them have been practically refurnished throughout.

Another costly and important improvement has been the erection of a central station for the distribution of light, heat and power throughout the hospital. In addition to these improvements, a model laundry of the very best design and equipment, an actino-therapeutic department, and an important extension of the casualty department have been provided.

The Nurses' Home.

Lastly Guy's is fortunate in the possession of one of the most beautiful of residences for its nurses in the Henriette Raphael Nurses' Home, which contains a large swimming-bath, and has been made beautiful by the benevolent givers, who expended a large sum of money upon marble and other embellishments. So remarkable is this home in an architectural and artistic sense that there was an inclination on the part of some critics to condemn it as too extravagant, until it was made clear that the entire buildings were provided and paid for by the Raphael family as a memorial to one of their beloved dead. In practice the Raphael Home has been a great boon. Indeed, it has proved an inspiration to many of the nursing staff, who have been fortunate to have had the privilege of residing within its walls.

It would have been a great work to have accomplished all we have just recorded in the circumstances named, but Mr. Cosmo Bonsor had to face an even more difficult task than the rebuilding and reconstruction of Guy's Hospital.

Splendid Finance.

At the time Mr. Bonsor entered upon the duties of treasurer at Guy's Hospital, one hundred and fifty of its beds were closed to the sick poor for want of funds, and, to make matters worse, as the late Mr. W. E. Gladstone pointed out, "its revenue, that was over 40,000*l*., had sunk to 20,000*l*. per annum." To meet this financial collapse, due to causes over which they could exercise no control, the Governors of Guy's Hospital resolved to entrust to Mr. Cosmo Bonsor the task of re-endowing Guy's Hospital to the extent of the loss of income sustained through the reduction of rental value. Our present King, then Prince of Wales, put himself at the head of this movement, and at a festival dinner at which he presided at the Imperial Institute, the largest sum ever raised for a hospital on such an occasion, £172,000 in round figures, was received and promised, including an additional revenue in annual subscriptions of £3,300 a year. That was a substantial send-off, and by persistent though quiet efforts the treasurer has obtained from voluntary sources during the twelve years he has held office a sum approaching three-quarters of a million sterling.

An Increased Income of £54,000 a Year.

The results of Mr. Cosmo Bonsor's management may be graphically shown in another way. Thus, the total income of Guy's Hospital for the twelve years ending March 31, 1895, was £510,821, or about £42,500 per annum. For the twelve years ending March 31, 1907, its total income from all sources in round figures was £1,160,000 or an average of nearly £97,000. Thus the average income of Guy's Hospital has increased under the present treasurer in twelve years by the enormous sum of £54,000 a year in round figures. Further and better still the re-endowment fund now amounts to £275,000 at least, the major portion of which is invested in approved securities, including Consols, bank and India stock, mortgages, freehold and leasehold property, colonial government and municipal securities, American gold bonds, debentures and debenture stock.

A Truly Splendid Achievement.

We believe we are justified in saying that never in the history of charity has a splendid work been better done than that which has been accomplished at Guy's Hospital during the treasurership of Mr. H. Cosmo Bonsor. Quietness and efficiency have made it the more praiseworthy, whilst the smallness of the cost, at which it has been accomplished, constitutes it a record performance in the history of hospital finance. We hope that some suitable and fitting recognition of Mr. Cosmo Bonsor's services may be speedily forthcoming, for they make him stand out as the first and most successful of hospital chairmen and treasurers.

